# Supplementary material for: Potential Benefits of Behaviors and Lifestyle for Human Health and Well-Being
Source: Nutrients. 2025 Oct 16;17(20):3253. doi: 10.3390/nu17203253 (PMC12566935; doi:10.3390/nu17203253)
Supplement: Supplementary file 1 [file nutrients-17-03253-s001.zip › nutrients-3893174-supplementary.pdf]

Supplementary Materials: Figure S1: KomPAN Questionnaire for the study of opinions and dietary habits for adolescents (16-18 years) and adults, version 2.2. – self-administered questionnaire).

KomPAN® Questionnaire for the study of opinions and dietary habits for adolescents (16-18 years) and adults
version 2.2 – self-administered questionnaire

We are conducting a scientific study in which we want to learn about the dietary habits of our university's academic community. The information provided is anonymous and confidential and will be used for scientific purposes only. Participation in the study is voluntary. Thank you in advance for your time and honest answers.

Please read the questions and mark one or more answers with an X, according to the instructions.

---

1. **Field of study (for students): …………………**

Date of study: **2. Day** |_|_| **3. Month** |_|_| **4. Year** |_|_|_|_|

Part A. Dietary Habits
We want to ask about your dietary habits over the **LAST YEAR**.

7. How many meals do you usually consume per day?
(1) __ 1 meal
(2) __ 2 meals
(3) __ 3 meals
(4) __ 4 meals
(5) __ 5 or more meals

Please recall ONE typical day in terms of your nutrition from the LAST WEEK and then answer the following questions.
18. What day of the week was it?
(You can select one answer)
(1) __ Monday
(2) __ Tuesday
(3) __ Wednesday
(4) __ Thursday
(5) __ Friday
(6) __ Saturday
(7) __ Sunday

19How many meals did you consume on that day?
Please enter the number …………………. meals on that day

20. How many times did you eat vegetables or fruit on that day?

Please enter the number …………………. times on that day

(Please include consumption during and between meals.)
21. Did you eat fast food on that day, e.g., French fries, hamburgers, pizza, hot dogs, toasted sandwiches?

(1) __ No
(2) __ Yes. How many times? Please enter the number …………………. times on that day

Part B. Food Consumption Frequency
When answering the questions, please consider food eaten during and between meals, at home and away from home, over the ***LAST YEAR****. In this section, please select* ***one answer*** *for each question.*

22. How often do you consume white bread, e.g., wheat, rye, mixed wheat-rye bread, toast bread, rolls, croissants?
(1) __ Never
(2) __ 1-3 times per month
(3) __ Once a week
(4) __ Several times a week
(5) __ Once a day
(6) __ Several times a day

23. How often do you consume wholemeal/wholegrain bread?
(1) __ Never
(2) __ 1-3 times per month
(3) __ Once a week
(4) __ Several times a week
(5) __ Once a day
(6) __ Several times a day

24. How often do you consume white rice, regular pasta, or fine-grain groats, e.g., semolina, couscous?
(1) __ Never
(2) __ 1-3 times per month
(3) __ Once a week
(4) __ Several times a week
(5) __ Once a day
(6) __ Several times a day

25. How often do you consume buckwheat groats, oats/oatmeal, whole-wheat pasta, or other coarse-grain groats?
(1) __ Never
(2) __ 1-3 times per month
(3) __ Once a week
(4) __ Several times a week
(5) __ Once a day
(6) __ Several times a day

26. How often do you consume fast food, e.g., French fries, hamburgers, pizza, hot dogs, toasted sandwiches?
(1) __ Never
(2) __ 1-3 times per month
(3) __ Once a week
(4) __ Several times a week
(5) __ Once a day
(6) __ Several times a day

27. How often do you consume fried dishes (e.g., meat or flour-based)?
(1) __ Never
(2) __ 1-3 times per month
(3) __ Once a week
(4) __ Several times a week
(5) __ Once a day
(6) __ Several times a day

28. How often do you consume butter as an addition to bread or dishes, for frying, baking, etc.?

(1) __ Never
(2) __ 1-3 times per month
(3) __ Once a week
(4) __ Several times a week
(5) __ Once a day
(6) __ Several times a day

29. How often do you consume lard as an addition to bread or dishes, for frying, baking, etc.?

(1) __ Never
(2) __ 1-3 times per month
(3) __ Once a week
(4) __ Several times a week
(5) __ Once a day
(6) __ Several times a day

31. How often do you consume milk (including flavored milk, cocoa, coffee with milk)?

(1) __ Never
(2) __ 1-3 times per month
(3) __ Once a week
(4) __ Several times a week
(5) __ Once a day
(6) __ Several times a day

32. How often do you consume fermented milk drinks, e.g., yogurts, kefirs (natural or flavored)?

(1) __ Never
(2) __ 1-3 times per month
(3) __ Once a week
(4) __ Several times a week
(5) __ Once a day
(6) __ Several times a day

33. How often do you consume quark / farmer's cheese (including homogenized cheese, quark desserts)?

(1) __ Never
(2) __ 1-3 times per month
(3) __ Once a week
(4) __ Several times a week
(5) __ Once a day
(6) __ Several times a day

34. How often do you consume hard/yellow cheese (including processed cheese, blue cheese)?

(1) __ Never
(2) __ 1-3 times per month
(3) __ Once a week
(4) __ Several times a week
(5) __ Once a day
(6) __ Several times a day

35. How often do you consume processed meats, sausages, or wieners?

(1) __ Never
(2) __ 1-3 times per month
(3) __ Once a week
(4) __ Several times a week
(5) __ Once a day
(6) __ Several times a day

36. How often do you consume dishes with so-called red meat, e.g., pork, beef, veal, mutton, lamb, game?

(1) __ Never
(2) __ 1-3 times per month
(3) __ Once a week
(4) __ Several times a week
(5) __ Once a day
(6) __ Several times a day

37. How often do you consume dishes with so-called white meat, e.g., chicken, turkey, rabbit?

(1) __ Never
(2) __ 1-3 times per month
(3) __ Once a week
(4) __ Several times a week
(5) __ Once a day
(6) __ Several times a day

38. How often do you consume fish?

(1) __ Never
(2) __ 1-3 times per month
(3) __ Once a week
(4) __ Several times a week
(5) __ Once a day
(6) __ Several times a day

40. How often do you consume dishes with legumes, e.g., beans, peas, soy, lentils?

(1) __ Never
(2) __ 1-3 times per month
(3) __ Once a week
(4) __ Several times a week
(5) __ Once a day
(6) __ Several times a day

42. How often do you consume fruit?

(1) __ Never
(2) __ 1-3 times per month
(3) __ Once a week
(4) __ Several times a week
(5) __ Once a day
(6) __ Several times a day

43. How often do you consume vegetables?

(1) __ Never
(2) __ 1-3 times per month
(3) __ Once a week
(4) __ Several times a week
(5) __ Once a day
(6) __ Several times a day

44. How often do you consume sweets, e.g., candies, cookies, cakes, chocolate bars, 'muesli' bars, other confectionery?

(1) __ Never
(2) __ 1-3 times per month
(3) __ Once a week
(4) __ Several times a week
(5) __ Once a day
(6) __ Several times a day

46. How often do you consume canned meat?

(1) __ Never
(2) __ 1-3 times per month
(3) __ Once a week
(4) __ Several times a week
(5) __ Once a day
(6) __ Several times a day

51. How often do you drink sweetened carbonated or non-carbonated beverages like Coca-Cola, Pepsi, Sprite, Fanta, etc.?

(1) __ Never
(2) __ 1-3 times per month
(3) __ Once a week
(4) __ Several times a week
(5) __ Once a day
(6) __ Several times a day

52. How often do you drink energy drinks, e.g., 2 KC, Black Horse, Red Bull, Burn, Shot, or others?
(1) Never
(2) 1-3 times per month
(3) Once a week
(4) Several times a week
(5) Once a day
(6) Several times a day

54. How often do you drink alcoholic beverages?
(1) Never
(2) 1-3 times per month
(3) Once a week
(4) Several times a week
(5) Once a day
(6) Several times a day

Part D. Lifestyle and Personal Data

In this section, we ask about your lifestyle and personal data. If you consider some questions too personal, you may refuse to answer. However, we will be grateful for every honest answer. In this section, please select **one answer for each question**.

80. Are you currently on any diet?
(1) __ No
(2) __ Yes, prescribed by a doctor for health reasons
(3) __ Yes, I am on a diet of my own choice

(Question for people currently on a diet)
81. Please specify the type of diet:
..
(Question for people currently on a diet)
82. How long have you been on the diet? Please enter the number: ....weeks, ...months,......... years.

83. How often do you eat out, e.g., in bars, restaurants, cafes, canteens?
(1) Never
(2) 1-3 times per month
(3) Once a week
(4) Several times a week
(5) Once a day
(6) Several times a day

(Question for people who drink alcoholic beverages)
84. What type of alcoholic beverage do you consume most frequently?
(1) __ Beer
(2) __ Wine
(3) __ Mixed drinks/Cocktails
(4) __ Spirits/Hard liquor

85. Do you currently smoke cigarettes, e-cigarettes, or use other nicotine products?
(1) __ No
(2) __ Yes

86. Have you smoked cigarettes, e-cigarettes, or used other nicotine products in the past?
(1) __ No
(2) __ Yes

87. On average, how many hours per day do you sleep on weekdays?
(1) __ 6 or less hours/day
(2) __ more than 6, but less than 9 hours/day
(3) __ 9 or more hours/day

88. On average, how many hours per day do you sleep on weekends?
(1) __ 6 or less hours/day
(2) __ more than 6, but less than 9 hours/day
(3) __ 9 or more hours/day

89. On average, how many hours per day do you spend watching TV or in front of a computer (including professional work)?
(1) __ less than 2 hours
(2) __ from 2 to almost 4 hours
(3) __ from 4 to almost 6 hours
(4) __ from 6 to almost 8 hours
(5) __ from 8 to almost 10 hours
(6) __ 10 hours or more

90. How do you assess your physical activity at work or school?
(1) __ Low: more than 70% of the time in a sitting position
(2) __ Moderate: about 50% of the time sitting and about 50% of the time in motion
(3) __ High: about 70% of the time in motion or physically demanding work

91. How do you assess your physical activity during your leisure time?
(1) __ Low: predominantly sitting, watching TV, reading, light housework, walking 1-2 hours a week
(2) __ Moderate: walking, cycling, gymnastics, gardening, or other light physical activity performed 2-3 hours a week
(3) __ High: cycling, running, gardening, or other recreational sports requiring physical effort performed over 3 hours a week

92. How do you assess your health status compared to other people of the same age?
(1) __ Worse than peers
(2) __ The same as peers
(3) __ Better than peers

93. How do you assess your nutritional knowledge?
(1) __ Insufficient
(2) __ Sufficient
(3) __ Good
(4) __ Very good

94. How do you assess your dietary pattern?
(1) __ Very poor
(2) __ Poor
(3) __ Good
(4) __ Very good

95. How do you assess your dietary pattern on weekdays compared to weekends?
(1) __ Essentially no difference
(2) __ Differs slightly
(3) __ Differs significantly

96. What is your body weight in kg? ____ kg
97. What is your height in cm? _____ cm
98. What is your waist circumference in cm?______ cm
If you do not know, please indicate the waist size you choose when buying clothes.

99. Sex:
(1) __ Male
(2) __ Female

100. Gender you identify with:
(1) __ The same
(2) __ Different
103. Year of birth: _____
104. What is your permanent place of residence?
(1) __ Village/Countryside
(2) __ City with less than 20,000 inhabitants
(3) __ City with 20,000 to 100,000 inhabitants
(4) __ City with more than 100,000 inhabitants
105. How many people are in your household (including yourself)? .... people
106. How many minors are in your household? .... people under 18
107. How do you assess your financial situation?
(1) __ Below average
(2) __ Average
(3) __ Above average

108. How do you assess your household's material status?
(1) __ We live very poorly – it's not enough even for basic needs
(2) __ We live modestly – we have to be very frugal on a daily basis
(3) __ We live on an average level – it's enough for daily life, but we have to save for major purchases
(4) __ We live well – we can afford a lot without special saving
(5) __ We live very well – we can afford a certain luxury
(Question for adults)
109. Are you professionally employed?
(1) __ No, I am retired or on a disability pension
(2) __ No, I am on parental leave, unemployed, or a homemaker
(3) __ Yes, but I work odd jobs/casually
(4) __ Yes, I have permanent employment
(5) __ No, I am a student
(Question for adults)
110. What is your education level?
(1) __ Primary
(2) __ Vocational
(3) __ Secondary (general or technical)
(4) __ Higher (Bachelor's, Engineer's, Master's degree)

Thank you very much for your time!

Supplementary Materials: Table S1: Mean response values (± standard deviation), medians, Mann-Whitney test results, and Glass Index (an indicator of effect size) for survey responses from staff and students. Question numbers marked in red (bold) indicate statistically significant differences between the staff and student groups.

Number of question	Students mean±SD	Employees mean±SD	Median for		Mann-Whitney test		r_g
			Students	Employees	U	p-value	
7	3.43±0.85	3.31±0.86	3	3	374	0.804	0.04
18	3.40±1.90	3.36±2.13	3	3	375	0.816	0.04
19	3.15±1.53	3.18±0.82	3	3	368	0.725	0.06
**20**	**1.30±1.17**	**2.08±1.40**	**2**	**1**	**252**	**0.027**	**-0.36**
21	1.00±0.40	1.15±0.26	1	1	328	0.325	-0.16
22	3.95±1.61	4.13±1.13	4	4	365	0.689	-0.07
23	2.45±1.39	2.82±1.43	3	2	335	0.383	-0.14
24	3.00±1.03	3.36±1.04	3	3	325	0.302	-0.17
25	2.60±1.14	2.56±1.07	2	2	388	0.981	0.01

**26**	**1.95±0.60**	**2.56±0.82**	**2**	**2**	**241**	**0.017**	**-0.38**
27	3.10±1.12	3.44±0.91	3	3	320	0.266	-0.18
28	3.55±1.67	3.62±1.48	4	4	384	0.923	-0.02
29	1.40±0.75	1.46±0.82	1	1	382	0.904	-0.02
31	3.35±1.73	3.31±1.44	3	4	388	0.981	0.01
32	3.85±1.39	3.32±1.42	3	4	293	0.157	0.23
33	3.35±1.23	3.08±1.33	3	3	329	0.329	0.16
34	3.60±1.19	3.85±1.20	4	4	349	0.511	-0.11
35	3.65±1.09	3.41±1.41	4	4	366	0.701	0.06
36	2.60±0.88	2.36±1.09	2	2	335	0.383	0.14
37	3.15±0.88	3.59±1.07	4	3	280	0.078	-0.29
38	2.35±0.59	2.13±0.78	2	2	308	0.242	0.19
40	2.25±0.72	2.05±0.96	2	2	320	0.330	0.16
42	4.35±1.09	4.05±1.21	4	4.5	332	0.437	0.13
43	4.40±0.82	4.55±1.03	4	4	353	0.665	-0.07
44	3.95±1.54	4.13±1.19	4	4	363	0.787	-0.04
46	1.35±0.49	1.24±0.43	1	1	337	0.487	0.11
**51**	**2.05±0.94**	**2.97±1.40**	**3**	**2**	**234**	**0.017**	**-0.38**
52	1.45±0.83	2.05±1.21	2	1	281	0.081	-0.28
54	2.03±0.83	1.82±0.68	2	2	349	0.511	0.11
80	1.40±0.82	1.46±0.82	1	1	372	0.779	-0.05
81	0.20±0.41	0.26±0.44	0	0	368	0.731	-0.06
82	0.50±1.15	0.28±0.51	0	0	382	0.904	-0.05
83	2.40±0.88	2.79±0.89	3	2	311	0.209	-0.20
84	1.50±1.19	1.99±1.60	1	1.5	333	0.361	-0.15
85	1.10±0.31	1.31±0.47	1	1	309	0.197	-0.21
86	1.25±0.44	1.41±0.50	1	1	328	0.321	-0.16
87	1.60±0.50	1.56±0.55	2	2	372	0.779	0.05
88	2.05±0.39	2.35±0.62	2	2	276	0.069	-0.29

89	2.60±1.14	3.21±1.44	3	2.5	303	0.166	-0.22
90	1.55±0.60	1.49±0.68	1	1.5	358	0.608	0.08
91	1.75±0.64	1.67±0.66	2	2	361	0.642	0.08
92	1.90±0.45	1.90±0.50	2	2	388	0.981	0.01
93	2.90±0.79	2.77±0.78	3	3	369	0.737	0.06
94	2.75±0.64	2.54±0.72	3	3	331	0.345	0.15
95	1.95±0.89	1.67±0.70	2	2	311	0.209	0.29
96	2.25±0.64	2.10±0.68	2	2	351	0.532	0.10
97	2.25±0.85	2.28±0.86	2	2	390	1.000	0.001
98	2.13±0.92	1.81±0.94	2	2	155	0.285	0.15
99	1.70±0.47	1.77±0.43	2	2	363	0.671	-0.07
100	1.05±0.22	1.03±0.16	1	1	381	0.885	0.02
**103**	**2.61±1.38**	**4.86±0.48**	**5**	**3**	**67**	**0.000002**	**-0.74**
**104**	**3.20±1.32**	**2.10±1.35**	**1**	**4**	**230**	**0.011**	**0.41**
**105**	**2.20±1.77**	**4.08±1.35**	**4**	**2**	**141**	**0.0001**	**-0.64**
106	0.25±0.72	0.51±0.64	0	0	284	0.091	-0.27
107	2.20±0.41	2.13±0.41	2	2	364	0.683	0.07
108	3.55±0.69	3.56±0.82	4	3	347	0.496	-0.11
**109**	**4.00±0.001**	**4.36±1.06**	**5**	**4**	**220**	**0.007**	**-0.44**
**110**	**3.90±0.31**	**3.31±0.47**	**3**	**4**	**159**	**0.0002**	**0.59**

Supplementary Materials: Table S2: Mean response values (± standard deviation), medians, Mann-Whitney test results, and Glass Index (rg, effect size index) for survey responses provided by women and men. Question numbers marked in red (bold) indicate statistically significant differences between the women and men.

Number of question	Female mean±SD	Male mean±SD	Median for		Mann-Whitney test		r_g
			Female	Male	U	p-value	
7	3.46±0.87	3.17±0.83	3	3	202	0,298	0.20
18	3.48±2.13	3.08±2.02	3	2,5	224	0,567	0.11
**19**	**3.38±1.13**	**2.58±1.00**	**3**	**3**	**145**	**0,027**	**0.42**
20	2.00±1.45	1.33±1.23	2	1	179	0,129	0.29

21	1.10±0.28	1.13±0.48	1	1	235	0,731	-0.07
22	4.19±1.40	3.75±1.06	4	4	205	0,333	0.19
23	2.57±1.40	2.75±1.42	2	2	228	0,625	-0.10
**24**	**3.38±1.01**	**2.67±0.98**	**4**	**2,5**	**153**	**0,040**	**0.39**
25	2.64±1.12	2.25±0.87	2	2	209	0,377	0.17
26	2.26±0.73	2.50±1.00	2	2,5	211	0,399	-0.16
27	3.24±0.96	3.50±1.24	3	4	218	0,486	-0.13
28	3.67±1.65	3.25±1.36	4	3,5	216	0,454	0.14
29	1.31±0.64	1.75±0.97	1	1,5	182	0,148	-0.28
31	3.38±1.43	2.75±1.54	3	2	190	0,197	0.25
32	3.59±1.30	3.25±1.66	4	3,5	215	0,510	0.13
**33**	**3.43±1.21**	**2.42±1.16**	**3**	**2**	**135**	**0,015**	**0.46**
34	3.90±1.12	3.33±1.23	4	3	188	0,186	0.25
35	3.29±1.37	3.92±0.79	4	4	187	0,176	-0.26
36	2.33±1.00	2.67±1.15	2	2,5	210	0,388	-0.17
37	3.50±0.89	2.92±1.24	4	3	183	0,151	0.28
38	2.29±0.81	2.00±0.45	2	2	180	0,268	0.22
40	2.10±0.82	2.18±1.08	2	2	230	0,991	0.004
42	4.33±1.10	3.82±1.40	4	4	174	0,211	0.24
43	4.67±1.00	4.00±0.77	4	4	156	0,102	0.32
44	4.12±1.38	3.82±1.17	4	4	204	0,554	0.12
46	1.24±0.43	1.36±0.50	1	1	202	0,532	-0.12
51	2.55±1.29	2.73±1.68	2	2	226	0,913	-0.02
52	1.83±1.19	1.75±1.06	1	1	247	0,925	0.02
54	1.77±0.61	2.25±1.14	2	2	196	0,248	-0.22
80	1.57±0.89	1.17±0.58	1	1	196	0,248	0.22
81	0.31±0.47	0.08±0.29	0	0	195	0,240	0.23
82	0.43±0.80	0.25±0.87	0	0	201	0,289	0.20
83	2.57±0.86	2.83±0.94	2	3	205	0,333	-0.19

84	1.86±1.52	1.67±1.56	2	1	231	0,662	0.09
85	1.19±0.40	1.33±0.49	1	1	216	0,460	-0.14
86	1.33±0.48	1.33±0.49	1	1	252	0,992	0
87	1.55±0.55	1.67±0.49	2	2	220	0,512	-0.13
88	2.29±0.51	2.17±0.72	2	2	230	0,655	0.09
89	2.98±1.28	2.58±1.51	3	2	201	0,289	0.20
**90**	**1.43±0.59**	**2.00±0.74**	**1**	**2**	**144**	**0,025**	**-0.43**
91	1.64±0.58	2.08±0.79	2	2	172	0,098	-0.32
92	1.93±0.46	1.83±0.58	2	2	230	0,647	0.09
93	2.88±0.77	2.75±0.75	3	3	221	0,526	0.12
94	2.67±0.69	2.58±0.79	3	3	250	0,967	0.01
95	1.74±0.73	1.75±0.97	2	2	249	0,950	-0.01
**96**	**1.98±0.56**	**2.67±0.78**	**2**	**2,5**	**135**	**0,015**	**-0.46**
**97**	**1.93±0.64**	**3.17±0.72**	**2**	**3**	**60**	**0,0001**	**-0.76**
98	1.83±0.97	2.33±0.87	2	2	89	0,159	-0.22
100	1.02±0.15	1.08±0.29	1	1	237	0,763	-0.06
103	4.08±1.42	3.92±1.44	5	5	213	0,733	0.06
104	2.50±1.40	2.00±1.48	3	1	206	0,344	0.18
105	3.50±1.80	3.08±1.83	4	3	225	0,581	0.11
106	0.43±0.63	0.50±0.90	0	0	249	0,959	0.01
107	2.14±0.42	2.17±0.39	2	2	247	0,925	-0.02
108	3.55±0.83	3.67±0.65	4	4	222	0,539	-0.12
109	4.29±0.71	3.92±1.38	4	4	225	0,581	0.11
110	3.55±0.50	3.50±0.52	4	3,5	240	0,811	0.05
